# Short-Term Esmolol Improves Coronary Artery Remodeling in Spontaneously Hypertensive Rats through Increased Nitric Oxide Bioavailability and Superoxide Dismutase Activity

**DOI:** 10.1155/2014/531087

**Published:** 2014-03-26

**Authors:** Ana Arnalich-Montiel, María Carmen González, Emilio Delgado-Baeza, María Jesús Delgado-Martos, Luis Condezo-Hoyos, Antonia Martos-Rodríguez, Pilar Rodríguez-Rodríguez, Begoña Quintana-Villamandos

**Affiliations:** ^1^Department of Anesthesiology, Reanimation and Intensive Care, Gregorio Marañón University General Hospital, 28007 Madrid, Spain; ^2^Department of Physiology, Faculty of Medicine, Autónoma University, 28029 Madrid, Spain; ^3^Department Experimental Medicine and Surgery, Gregorio Marañón University General Hospital, 28007 Madrid, Spain; ^4^Department of Pharmacology, Faculty of Medicine, Complutense University, 28040 Madrid, Spain

## Abstract

The aim of this study was to assess the effects of short-term esmolol therapy on coronary artery structure and function and plasma oxidative stress in spontaneously hypertensive rats (SHR). For this purpose, 14-month-old male SHR were treated for 48 hours with esmolol (SHR-E, 300 **μ**g/kg/min). Age-matched untreated male SHR and Wistar Kyoto rats (WKY) were used as hypertensive and normotensive controls, respectively. At the end of intervention we performed a histological study to analyze coronary artery wall width (WW), wall-to-lumen ratio (W/L), and media cross-sectional area (MCSA). Dose-response curves for acetylcholine (ACh) and sodium nitroprusside were constructed. We also assessed several plasma oxidative stress biomarkers, namely, superoxide scavenging activity (SOSA), nitrites, and total antioxidant capacity (TAC). We observed a significant reduction in WW (*P* < 0.001), W/L (*P* < 0.05), and MCSA (*P* < 0.01) and improved endothelium-dependent relaxation (AUC_SHR-E_ = 201.2 ± 33 versus AUC_SHR_ = 97.5 ± 21, *P* < 0.05) in SHR-E compared with untreated SHR; no differences were observed for WW, MCSA, and endothelium-dependent relaxation by ACh at higher concentrations (10^−6^ to 10^−4^ mol/l) for SHR-E with respect to WKY. SOSA (*P* < 0.001) and nitrite (*P* < 0.01) values were significantly higher in SHR-E than in untreated SHR; however, TAC did not increase after treatment with esmolol. Esmolol improves early coronary artery remodeling in SHR.

## 1. Introduction

The risk of fatal cardiovascular events is associated with adverse structural and functional remodeling of the vasculature, which is frequently caused by hypertension [1]. Regression of these changes is a goal of antihypertensive therapy [2] and is associated with reduced incidence of cardiovascular events [3, 4]. Chronic treatment with antihypertensive agents (angiotensin-converting enzyme inhibitors, angiotensin II receptor blockers, calcium antagonists, *β*-blockers, and diuretics) has led to regression of vascular remodeling both in humans and in animal models of hypertension [2, 5, 6]. However, early regression of coronary artery remodeling after short-term therapy with these agents has not been investigated.

Esmolol is an ultrashort-acting cardioselective *β*-adrenergic blocker with a half-life of ~2 minutes, a time to peak effect of about 6–10 minutes, and a washout time of 9 minutes [7]. Therefore, esmolol is faster acting than other *β*-blockers and is an excellent option for the treatment of arterial hypertension and undesirable increases in heart rate [8], although its effect on vascular remodeling has not been established.

Our group previously demonstrated that short-term treatment (48 hours) with esmolol reduces left ventricular hypertrophy in spontaneously hypertensive rats (SHR) [9]. However, early changes in vascular remodeling and oxidative stress following short-term use of this agent have not been analyzed to date.

In the present study, we hypothesized that even short-term administration (48 hours) of esmolol could reduce structural and functional coronary artery remodeling by increasing the bioavailability of nitric oxide (NO) and superoxide dismutase activity (SOD) in SHR. Therefore, we analyzed the protective effect of esmolol in coronary arteries and antioxidant status in plasma.

## 2. Methods

All procedures conformed to the Guide for the Care and Use of Laboratory Animals (NIH publication number 85-23, revised in 1996) and Spanish legislation (RD 1201/2005) and were approved by the Ethics Committee of Hospital General Universitario Gregorio Marañon, Madrid, Spain.

### 2.1. Experimental Design

The study animals—14-month-old male SHR (*n* = 22) and normotensive control Wistar-Kyoto (WKY) rats (*n* = 11)—were bred at the animal house of Universidad Autónoma de Madrid. All the rats were supplied with standard rat chow and drinking water ad libitum and were maintained on a 12 h/12 h light/dark cycle. The animals were housed at a constant temperature of 24°C and relative humidity of 40%. All the rats were anesthetized with an intraperitoneal injection of diazepam 4 mg/kg/1 and ketamine 10 mg/kg/1, and a catheter was inserted into the right internal jugular vein under sterile conditions. SHR were randomly divided into 2 groups (11 rats each): rats treated with esmolol (SHR-E) and rats treated with vehicle (SHR, hypertensive control group). SHR-E received an intravenous infusion of esmolol at 300 *μ*g/kg/min for 48 hours. SHR and WKY received saline solution (vehicle). After 48 hours of treatment, the rats were killed by decapitation. Blood samples and the heart were removed immediately to study oxidative stress and perform structural and vascular reactivity experiments.

### 2.2. Blood Pressure and Heart Rate Measurements

Systolic arterial pressure (SAP) and heart rate (HR) were measured (conscious animals prewarmed to 35°C in thermostatic cages) by the tail-cuff method with a photoelectric sensor (Niprem 546, Cibertec, Madrid, Spain). Several determinations were made and the findings were considered valid if 10 consecutive measurements were within 10 mmHg of each other.

### 2.3. Morphology of Intramyocardial Arteries

We analyzed 5 animals from each group. Left ventricular tissue was fixed in 4% sodium-buffered formaldehyde. We made sequential transversal cuts of the left ventricle from the subvalvular region to the apex. Samples were then dehydrated and embedded in paraffin. Serial sections (5 *μ*m) were stained with orcein. We differentiated between the subepicardial and midmyocardial parts of the left ventricular wall, following the procedure described by Lunkenheimer et al. [10]. We then located the intramyocardial branch of the obtuse marginal artery (branch of the circumflex coronary artery). A total of 30 intramyocardial arteries (2 vessels per rat) were observed and analyzed using a high-resolution camera (Sony CCD IRIS) attached to a microscope (Leica DMLB, ×40). The morphometric analyses were performed using the method of Gundersen el al. [11]. The images were projected on a computer screen, and the external diameter (ED) (inner diameter + tunica intima + tunica media) and lumen diameter (LD) of the coronary arteries were measured. The wall width (WW) was expressed as (ED-LD)/2. The wall-to-lumen ratio (W/L) was expressed as (WW/LD) × 100, and the media cross-sectional area (MCSA) (tunica intima + tunica media) was expressed as (*π*/4) × (ED^2^−LD^2^) [6].

### 2.4. Blood Collection and Plasma Preparation

We analyzed 6 animals from each group. Blood (2.4 mL) was collected from each animal in Vacutainer tubes (BD, Plymouth, UK) containing citrate (300 *μ*L). Blood samples were centrifuged at 900 g for 10 minutes at 4°C to obtain plasma, which was aliquoted and stored at –80°C for further analysis.

#### 2.4.1. Nitrite

The plasma nitrite level was assessed using a Griess reaction-based protocol adapted from Giustarini et al. [12] and Miranda et al. [13]. One hundred microliters of plasma or nitrite standard (0–100 *μ*M) was mixed with 10 *μ*L de N-ethylenediamine (150 mM) to eliminate thiols that interfere with the Griess reaction. Thereafter, the protein from samples was precipitated by addition of 110 *μ*L of trichloroacetic acid (20% w/v) and incubation in an ice bath for 5 minutes. Fifty microliters of supernatant obtained by centrifugation at 12,000 g for 5 minutes (4°C) was mixed on 96-well plate with 50 *μ*L of saturated vanadium chloride (dissolved in HCl 1 M), 25 *μ*L of sulphanilamide (2% w/v in HCl 5% v/v), and 25 *μ*L of N-naphthyl-ethylenediamine (0.1% w/v in water). Finally, the mixture was incubated at 37°C for 1 hour, and the absorbance was read at 540 nm. Plasma nitrite content was expressed in *μ*M.

#### 2.4.2. Quantification of Superoxide Scavenging Activity (SOSA)

Plasma superoxide anion scavenging activity was assessed using the SOSA assay based on the inhibition of luminescence emitted by coelenterazine, which is oxidized by superoxide anion [14]. Superoxide anion was produced using a hypoxanthine/xanthine oxidase system. SOSA values were quantified by comparing the luminescence inhibition of each sample with that observed on the superoxide dismutase activity standard curve (0–4 mU/mL) and expressed as mU/mL.

#### 2.4.3. Catalase Activity

Catalase activity was assessed using the Amplex red catalase assay (Catalase Assay Kit with Amplex Ultra Red reagent; Invitrogen). Catalase activity was expressed as units per milligram of protein.

#### 2.4.4. Total Antioxidant Capacity (TAC)

TAC was assessed using a modified CUPRAC-BCS assay. Briefly, 10 *μ*L of diluted plasma with phosphate buffer (10 mM, pH = 7.4) was mixed on 96-well plate with 190 *μ*L of BCS 0.25 mM (dissolved in phosphate buffer), and the initial absorbance (Ai) was measured at 490 nm. Thereafter, 50 *μ*L of CuSO_4_ (0.5 mM dissolved in water) was added, the mix was incubated in darkness at room temperature for 5 minutes, and the final absorbance (Af) was measured at 490 nm. TAC was calculated from (Af-Ai) and the standard curve and expressed as mM Trolox.

#### 2.4.5. Total Thiols

Plasma thiols were assessed using the microplate 5,5′-dithiobis (2-nitrobenzoic acid) assay [15]. The absorbance was measured at 412 nm in a Synergy HT Multi-Mode Microplate Reader (BioTek, Potton, UK), and thiol content was expressed as millimoles per liter of reduced glutathione per milligram of protein.

#### 2.4.6. Protein Content

Protein content was assessed using a Coomassie blue-based microtiter plate assay with bovine serum albumin as standard (Bio-Rad). The absorbance was measured at 595 nm in a Synergy HT Multi-Mode Microplate Reader.

### 2.5. Vascular Reactivity of Coronary Arteries

We analyzed 6 animals from each group. The heart was removed and maintained in cold (4°C) oxygenated Krebs-Henseleit solution (KHS, in mmol·L^−1^: 115 NaCl, 25 NaHCO_3_, 4.7 KCl, 1.2 MgSO_4_·7H_2_O, 2.5 CaCl_2_, 1.2 KH_2_PO_4_, 11.1 glucose, and 0.01 Na_2_EDTA). Segments of the left anterior descending artery were isolated, and surrounding cardiac tissue was cleaned under a dissecting microscope. Segments of coronary arteries (2 mm in length) were mounted on a wire myograph (Multi Myograph System, model 610 M; Danish Myo-Technology) coupled to a Powerlab data acquisition system (AD-Instruments, Castle Hill, Australia) and studied as described elsewhere [16]. Briefly, the vessel was mounted in oxygenated KHS. The arteries were then set to a normalized internal circumference of 0.9 L100, which was considered as the effective lumen diameter that represented the basal tone of the artery. After an equilibration period in KHS at 37°C and pH 7.4, segments were stretched to their optimal lumen diameter to develop active tension. Coronary arteries were exposed to 120 mmol·L^−1 ^K^+^-KHS in order to ensure their functional integrity. Responses to acetylcholine (ACh, 10^−9^ to 10^−4 ^mol/l) were studied in segments precontracted with 5-hydroxytryptamine (5-HT, 3 × 10^−7 ^mol/l). After a washout period of 60 minutes, dose-response curves were constructed for sodium nitroprusside (SNP, 10^−9^ to 10^−4 ^mol/l) to study endothelium-independent relaxation in segments precontracted with 5-HT (3 × 10^−7^ mol/l).

### 2.6. Data Analysis and Statistics

The results were expressed as mean ± SEM. Between-group comparisons were made by 1-way analysis of variance followed by a Bonferroni post hoc test. Relaxing responses are expressed as percentage reduction in the 5-HT preconstricted state. To compare the effect of vasodilator drugs on response to 5-HT in coronary segments, some results were expressed as differences in the area under the concentration-response curves between the 3 experimental groups. *P* < 0.05 was considered statistically significant. The statistical analysis was performed using SPSS 17.0 for windows (SPSS, Chicago, Illinois, USA) and S-PLUS 6.1.

## 3. Results

### 3.1. Physiological Parameters

Body weight was higher in WKY than in SHR (441.60 ± 22.08 g versus 405.13 ± 22.12 g, *P* < 0.05) and SHR-E (441.60 ± 22.08 g versus 400.15 ± 11.07 g, *P* < 0.05). Heart weight was higher in SHR than in WKY (2.10 ± 0.27 g versus 1.51 ± 0.11 g, *P* < 0.05) but decreased after treatment in SHR-E compared with SHR (1.49 ± 0.12 g versus 2.10 ± 0.27 g, *P* < 0.05). There were no significant differences in heart weigh between SHR-E and WKY.

### 3.2. Hemodynamic Parameters

SAP was higher in SHR than in WKY (236 ± 1.5 versus 135 ± 0.1, *P* < 0.001). After 48 hours of treatment, esmolol lowered SAP in SHR-E with respect to SHR (149 ± 2 versus 236 ± 1.5, *P* < 0.001), and the SAP values at the end of treatment were not comparable with those of the WKY (*P* < 0.05). Heart rate remained unchanged in both SHR and WKY (297 ± 1 versus 297 ± 2) but decreased after treatment in SHR-E compared with SHR (183 ± 3 versus 297 ± 1, *P* < 0.001) and WKY (*P* < 0.001).

### 3.3. Effect of Esmolol on Intramyocardial Artery Morphology

**Figure 1:**
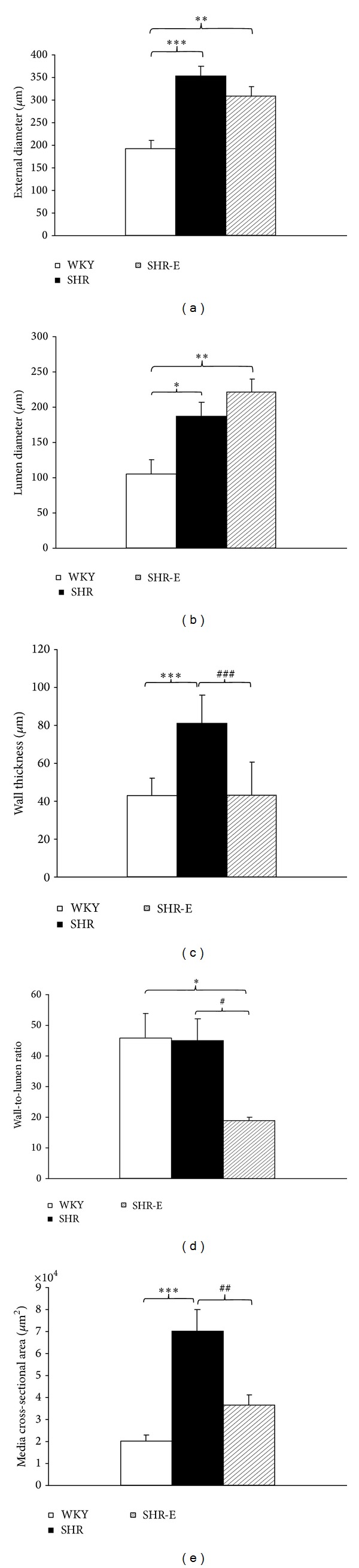
Structural parameters. (a) External diameter, (b) lumen diameter, (c) wall thickness, (d) wall-to-lumen ratio, and (e) media cross-sectional area of intramyocardial artery of the left ventricle from Wistar Kyoto control rats (WKY, *n* = 5), spontaneously hypertensive control rats (SHR, *n* = 5), and spontaneously hypertensive rats treated with esmolol (SHR-E, *n* = 5). Data are expressed as mean ±SEM. Statistically significant differences between WKY, SHR, and SHR-E are shown (*P < 0.05 versus WKY; **P < 0.01 versus WKY; ***P < 0.001 versus WKY; ^#^ *P* < 0.05 versus SHR; ^##^ *P* < 0.01 versus SHR; ^###^ *P* < 0.001 versus SHR).

**Figure 2:**
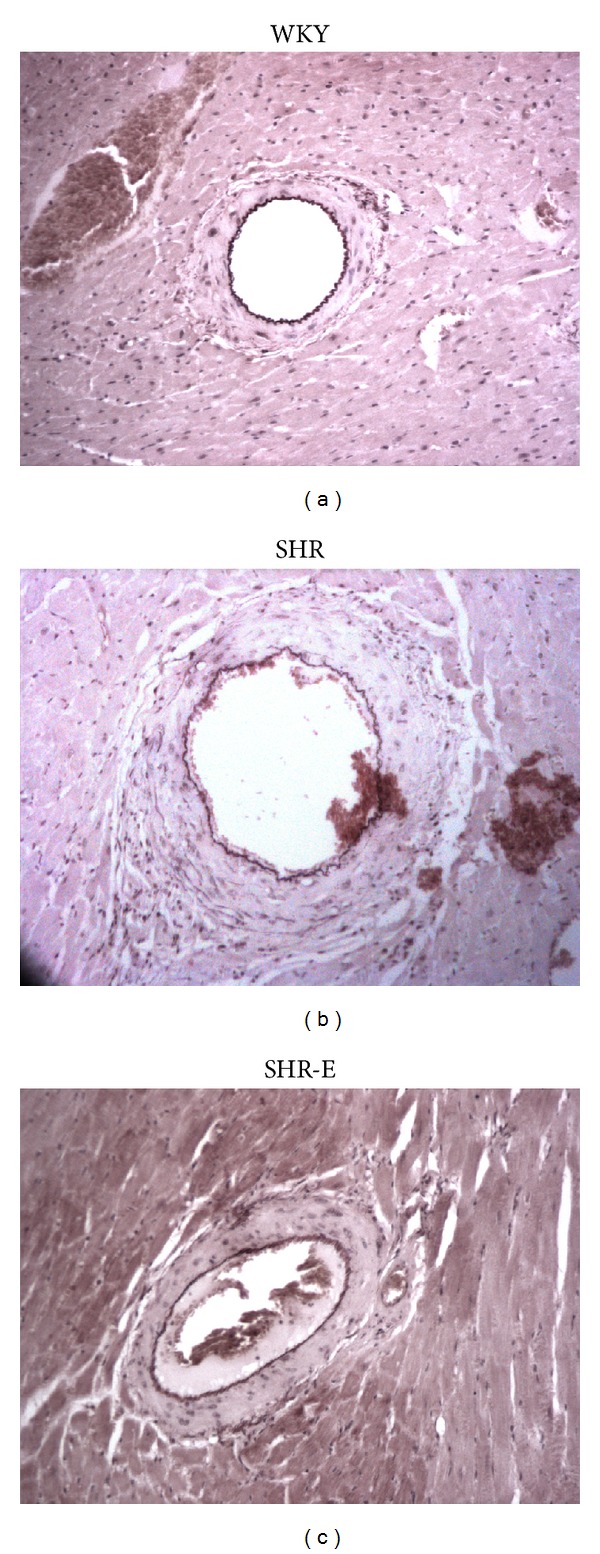
Examples of histological sections of the intramyocardial artery of the left ventricle from a Wistar Kyoto control rat (WKY), spontaneously hypertensive control rat (SHR), and spontaneously hypertensive rat treated with esmolol (SHR-E). Orcein x10.

Intramyocardial artery ED was significantly greater in SHR than in WKY. Administration of esmolol to SHR-E decreased ED, although the difference was not statistically significant compared with untreated SHR (Figures 1(a) and 2). The LD of the artery in SHR was significantly greater than in WKY. Administration of esmolol increased LD by a greater amount in SHR-E than in SHR, although the difference was not statistically significant (Figures 1(b) and 2). The WW of the artery was significantly higher in SHR than in WKY. Interestingly, WW was significantly lower after 48 hours of treatment in SHR-E than in SHR, although no differences were detected with respect to WKY (Figures 1(c) and 2). The W/L in SHR did not differ from that in the WKY. Administration of esmolol significantly decreased the W/L ratio in SHR-E (Figures 1(d) and 2). The MCSA in the SHR was larger than in the WKY. Interestingly, MCSA was significantly lower after 48 hours of treatment in SHR-E than in SHR; no differences were observed with respect to WKY (Figures 1(e) and 2).

### 3.4. Effect of Esmolol on Coronary Artery Vasodilator Function

**Figure 3:**
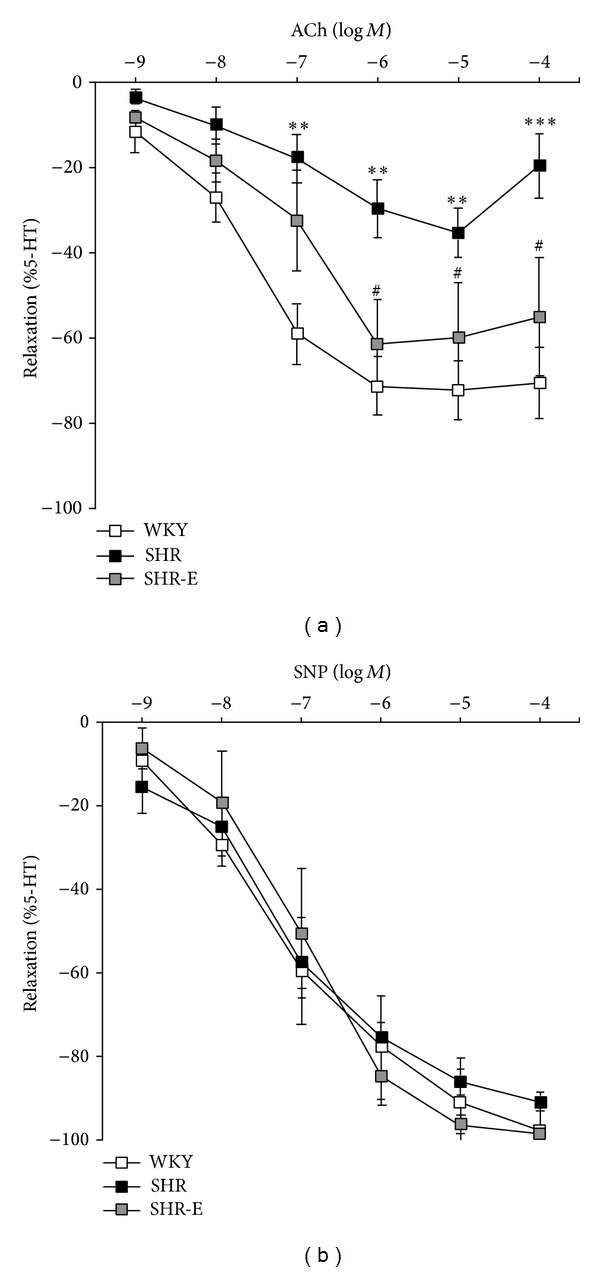
Vascular reactivity parameters. (a) acetylcholine (ACh) responses and (b) sodium nitroprusside (SNP) in left anterior descending artery precontracted with 5-hydroxytryptamine (5-HT, 3 × 10^−7 ^mol/l) from Wistar Kyoto control rats (WKY, *n* = 6), spontaneously hypertensive control rats (SHR, *n* = 6), and spontaneously hypertensive rat treated with esmolol (SHR-E, *n* = 6). Relaxing responses are expressed as percentage reduction in the 5-HT preconstricted state. Data are expressed as mean ± SEM. Statistically significant differences between WKY, SHR, and SHR-E are shown (**P < 0.01 versus WKY; ***P < 0.001 versus WKY; ^#^ *P* < 0.05 versus SHR).

In WKY and SHR-E rats, ACh elicited concentration-dependent relaxation at all the concentrations tested, whereas in SHR, ACh elicited a biphasic response with dilatation at low concentrations (10^−9^ to 10^−5 ^mol/l) and contractions at higher concentrations (10^−4 ^mol/l). The endothelium-dependent relaxation induced by ACh in 5-HT contracted coronary arteries was significantly lower in SHR than in WKY (10^−7^ to 10^−4 ^mol/l), and esmolol significantly improved this relaxation (10^−6^ to 10^−4^). Treatment with esmolol normalized coronary artery endothelium-dependent relaxation by ACh at higher concentrations (10^−6^ to 10^−4 ^mol/l) (Figure 3(a)). The AUC was significantly larger in WKY than in SHR (AUC_WKY_ = 265.9 ± 27 versus AUC_SHR_ = 97.5 ± 21, *P* = 0.0002). AUC was significantly higher in SHR-E than in SHR (AUC_SHR*-*E_ = 201.2 ± 33 versus AUC_SHR_ = 97.5 ± 21, *P* = 0.027); no differences were observed for SHR-E with respect to WKY.

No differences in the concentration-response curves for the endothelium-independent vasodilator SNP (10^−9^ to 10^−4 ^mol/l) were observed between the 3 experimental groups (AUC_WKY_ = 292.9 ± 34; AUC_SHR_ = 253.2 ± 19; AUC_SHR*-*E_ = 307.2 ± 34) (Figure 3(b)). Maximal dilatation to SNP was 97.7 ± 1% in WKY, 91.4 ± 2% in SHR, and 98.6 ± 1% in SHR-E (Figure 3(b)).

### 3.5. Effect of Esmolol on NO Level and Plasma Antioxidant Status

**Figure 4:**
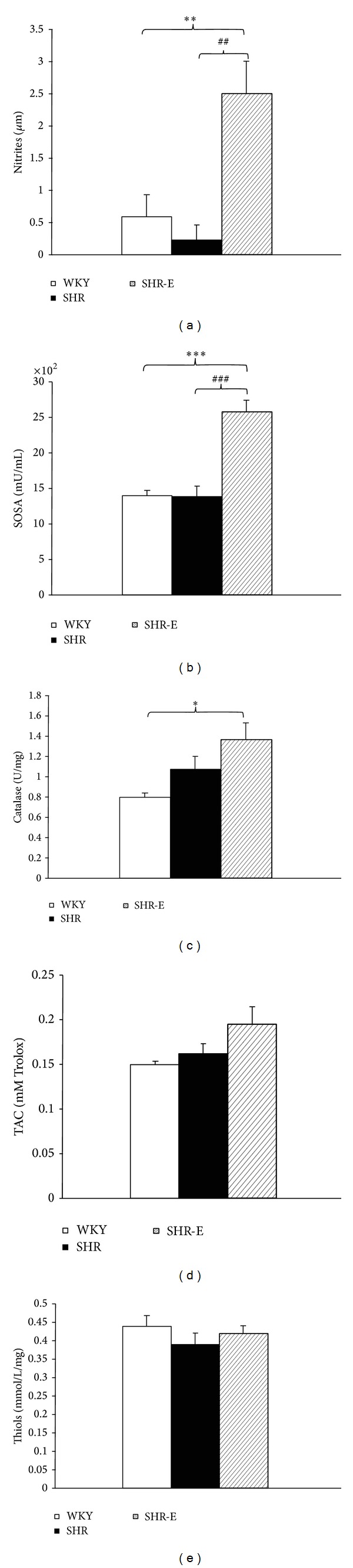
Antioxidant parameters. (a) Nitrites, (b) superoxide scavenging activity (SOSA), (c) catalase, (d) total antioxidant capacity (TAC), and (e) thiols on plasma from Wistar Kyoto control rats (WKY, *n* = 6), spontaneously hypertensive control rats (SHR, *n* = 6), and spontaneously hypertensive rat treated with esmolol (SHR-E, *n* = 6). Data are expressed as mean ± SEM. Statistically significant differences between WKY, SHR, and SHR-E are shown (*P < 0.05 versus WKY; **P < 0.01 versus WKY; ***P < 0.001 versus WKY; ^##^ *P* < 0.01 versus SHR; ^###^ *P* < 0.001 versus SHR).

Interestingly, plasma nitrite level was significantly higher after 48 hours of treatment in SHR-E than in SHR (Figure 4(a)). No differences were observed between SHR and WKY, although a trend toward reduction was recorded (Figure 4(a)). Moreover, the SOSA value, which is in part related to superoxide dismutase activity, was significantly increased after treatment with esmolol in SHR-E, although no differences were observed for this enzyme in SHR compared with WKY (Figure 4(b)). Similarly, administration of esmolol increased catalase activity in SHR-E but not in SHR or WKY (Figure 4(c)). Finally, administration of esmolol did not change total plasma antioxidant activity (Figure 4(d)) or thiol level (Figure 4(e)) in SHR-E, SHR, or WKY.

## 4. Discussion

Our results show that short-term (48 hours) intravenous infusion of esmolol (300 *μ*g/kg/min) in adult SHR induces early changes of coronary artery remodeling by increased bioavailability of NO and improved antioxidant status in plasma. This is the first study to show an association between early improvement in coronary artery remodeling with short-term administration of a *β*-blocker. Although previous human and experimental animal studies have demonstrated that various antihypertensive drugs can reverse coronary artery remodeling, they have all been conducted with drugs administered over several months [17–22].

### 4.1. Effect of Esmolol on Vascular Structure and Function

Hypertension is associated with structural and functional changes in vascular remodeling, which in turn increase cardiovascular risk [23]. In our experiments, we found that WW, MCSA, and LD of intramyocardial arteries in 14-month-old SHR were higher than in WKY. These data are consistent with those of Cebova and Kristek [24], who reported remodeling in the septal branch of the descending coronary artery in 13-month-old SHR; however, the authors found no differences in young SHR and age-matched WKY. The gradual increase in all 3 parameters (WW, MCSA, and LD) with age in coronary artery hypertrophy in SHR could reflect an adaptive mechanism triggered by chronic arterial hypertension.

At 14 months of age, SHR present compensated left ventricular hypertrophy (LVH) [25], which is associated with functional and structural alterations of the coronary artery [26]. We previously reported that 48-hour treatment of SHR with esmolol produces regression of LVH [9], and the results of the present study suggest that coronary artery WW and MCSA in SHR normalizes to those of WKY after treatment. These data are consistent with the effect of other antihypertensive agents (losartan [17], perindopril [18], amlodipine and enalapril [19], indapamide [20], and carvedilol [22]) in coronary artery remodeling, although only after long-term treatment. Regression of vascular remodeling is difficult, even with long-term pharmacological treatment [27], although esmolol produced changes in vascular structure and function in 48 hours. This effect may be attributed in part to the fact that short-term intravenous administration of esmolol exerts a greater hypotensive effect than other drugs [28]. We do not know whether esmolol plays a role in fibrosis, hypertrophy, or both in the coronary arteries. However, in a previous study [9], we showed that esmolol (300 *μ*g/kg/min for 48 h) produces early changes in cross-sectional area of left ventricular cardiomyocytes (in subepicardial region) in SHR but does not affect collagen volume fraction.

Our results show hypertrophic outward remodeling (associated with an increase in arterial wall mass and lumen thickness) [1] in intramyocardial arteries in 14-month-old SHR. However, esmolol produced eutrophic outward remodeling (decreases in wall thickness to inner diameter ratio), which is associated with a decrease in vascular resistance and, consequently, a decrease in arterial pressure [29].

### 4.2. Effect of Esmolol on Plasma Antioxidant Parameters

Esmolol is an ultrashort-acting specific blocker of *β*1-adrenergic receptors with an elimination half-life of 9 minutes [30]; in other words, recovery from *β* blockade (i.e., heart rate approaching baseline levels) can be achieved within 10 minutes of discontinuing the infusion [31]. We recently demonstrated that short-term treatment with esmolol can reverse early left ventricular hypertrophy in the SHR model of stable compensated ventricular hypertrophy [9], although the underlying mechanism has not been explored. The level of plasma nitrite in SHR-E was significantly higher than in the SHR and WKY groups. Nevertheless, a similar improvement has been reported for other blockers of *β*-adrenergic receptors, such as nebivolol [32], carvedilol [33], and nipradilol [34]. The increase in plasma NO level after administration of esmolol to SHR was markedly greater than with *β*1-adrenergic receptor blockers, even in WKY [32]. On the other hand, the reduced asymmetric dimethylarginine (ADMA) concentration in SHR treated with nebivolol, a third-generation *β*-blocker, has been associated with endothelial NO synthase (eNOS) agonist properties and increased bioavailability of NO [32]. In addition, ADMA has been shown to increase ROS level in arterioles in vitro by activation of the vascular renin-angiotensin system [35]. In our study, we showed that SOSA, which includes low-molecular weight antioxidant (scavengers) and superoxide dismutase, increased as a result of treatment with esmolol. The fact that total antioxidant activity does not increase after treatment with esmolol reflects an increase in superoxide dismutase activity measured using the SOSA assay. Likewise, catalase activity was also increased by esmolol in SHR. Esmolol does not exert its antioxidant activity through ROS-scavenging mechanisms [33] similar to those found in this study, and total antioxidant activity did not change with esmolol, although plasma superoxide dismutase activity and catalase activity can be induced by esmolol. This explains how the increase in plasma NO level in SHR after treatment with esmolol was markedly greater than with other *β*-blockers. Based on these results, esmolol can improve the oxidative stress associated with arterial hypertension. Antioxidant capacity is one of the mechanisms that underlie the efficacy of antihypertensive therapy [36].

Esmolol increases nitric oxide bioavailability and superoxide dismutase activity. These effects could explain structural changes and attenuation of coronary artery dysfunction. However, the vascular remodeling (structural changes) in hypertension is also associated with activation of the renin-angiotensin system, endothelin-1, endothelial dysfunction, oxidative stress, and ADMA [37].

### 4.3. Study Limitations

This study was designed to analyze the effect of esmolol on the structure and function of the coronary arteries. Although esmolol produces changes in coronary artery structure and function, we cannot conclude that regression of vascular remodeling occurred, because we did not study aspects such as markers of smooth muscle proliferation, activity of matrix metalloproteinases, and staining of perivascular fibrosis. Future studies will be necessary to answer this question.

In conclusion, our results show that esmolol improves coronary artery remodeling by increasing bioavailability of NO and improving antioxidant status in plasma in SHR, because lower WW and lower MCSA, improved endothelial dysfunction, and increased plasma NO level and superoxide dismutase activity were observed in SHR-E compared with the control SHR. Ours is the first study to show improvement of coronary artery remodeling with a *β*-blocker in the short term. If these results are confirmed in humans, esmolol could be taken into consideration for the treatment of patients with coronary artery disorders caused by arterial hypertension.
